# Orthopedic Rehabilitation Following Complex Repair of the Flexor and Extensor Tendons, Artery, and Nerve: A Case Report

**DOI:** 10.7759/cureus.99643

**Published:** 2025-12-19

**Authors:** Aydan Novruzova

**Affiliations:** 1 Physical Medicine and Rehabilitation, Kocaeli University, Kocaeli, TUR

**Keywords:** complex hand injury, early and controlled rehabilitation, mid-position splint, orthopedic rehabilitation, tendon repair

## Abstract

Simultaneous injury of both the flexor and extensor tendons of the hand is a rare condition that can lead to significant functional impairment and is often accompanied by arterial and nerve damage. This study aims to share the clinical outcomes of the rehabilitation process following a complex hand injury. Surgical repair was performed on a patient with injuries to the flexor and extensor tendons, as well as the radial artery and median nerve, followed by an intensive and structured rehabilitation program. The rehabilitation protocol was initiated with early passive movements and later progressed to include controlled active exercises, sensory training, and muscle strengthening. Functional outcomes were evaluated over a short-term follow-up period of 12 weeks using objective clinical measures, including range of motion and grip strength. Although validated functional outcome scales such as the Disabilities of the Arm, Shoulder, and Hand or Patient-Rated Wrist Evaluation were not applied, meaningful improvements in functional hand use were observed. This case highlights that in situations where tendon, nerve, and arterial injuries occur concurrently, early and controlled rehabilitation plays a critical role in functional recovery alongside surgical treatment. However, long-term follow-up is required to fully evaluate nerve regeneration and sustained functional outcomes.

## Introduction

The hand is highly susceptible to trauma due to its complex anatomy and fine functional characteristics. Injuries sustained in daily life or occupational settings, such as lacerations, punctures, or crush injuries, can simultaneously affect tendons, nerves, and vascular structures [1]. Flexor and extensor tendon injuries are particularly important for the preservation of overall hand function. Flexor tendon injuries carry a high risk of adhesions, rupture, or restricted motion, whereas extensor tendon injuries can result in significant impairments in grip and fine motor skills [2,3]. The functional recovery process becomes even more challenging in cases accompanied by nerve injuries. Median nerve damage, in particular, may lead to sensory deficits on the palmar aspect of the hand and impaired fine motor functions [4]. Additionally, vascular injuries, especially radial artery damage, directly influence the success of surgical repair and the course of subsequent rehabilitation. Maintaining adequate circulation, controlling edema, and ensuring proper wound healing play a critical role in this process.

The literature emphasizes that a multidisciplinary approach and individualized rehabilitation programs enhance functional recovery in complex hand injuries [5,6]. In cases where tendon, nerve, and arterial injuries coexist, postoperative rehabilitation is as crucial as surgical repair in restoring functional hand use. This case report details the rehabilitation process following the surgical repair of the flexor and extensor tendons, radial artery, and median nerve in a single hand.

## Case presentation

A 38-year-old, right-hand-dominant female patient with no known comorbidities, working in an office-based occupation, presented with a laceration injury to the right (dominant) hand caused by glass, involving both the palmar and dorsal surfaces in Zones II and III. On clinical examination, there was loss of flexion and extension in the second and third fingers, absence of radial artery pulse, hypoesthesia in the median nerve distribution, and edema in the hand and fingers. The diagnosis was primarily based on physical examination and palpation findings. Both flexor and extensor tendon injuries were identified in the second and third digits, involving the flexor digitorum profundus, flexor digitorum superficialis, and extensor digitorum communis tendons.

Emergency surgical intervention was performed by the orthopedics department. Flexor tendon injuries were repaired using the modified Kessler technique, while extensor tendons were treated with a four-strand core repair. The radial artery was reconstructed with an end-to-end microsurgical anastomosis, and the median nerve was repaired with an epineural suture. Postoperative vascular status was clinically monitored through capillary refill time, skin temperature, and color assessment, with no signs of vascular compromise observed during follow-up. Due to the stable clinical findings, additional imaging studies for arterial patency were not deemed necessary.

**Figure 1:**
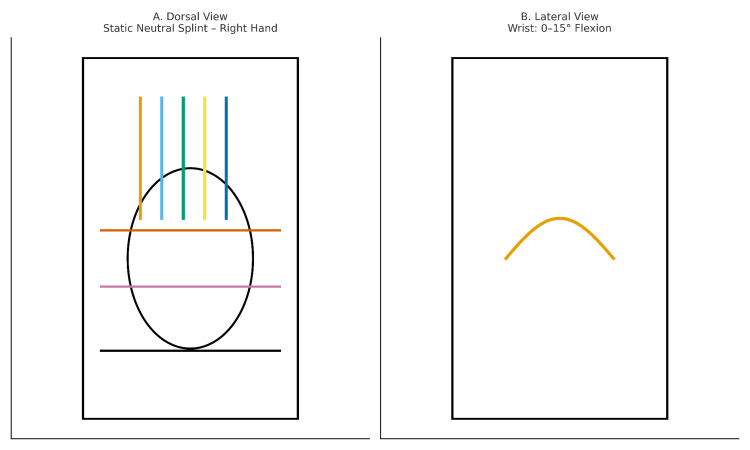
Dorsal (A) and lateral (B) schematic views of the static neutral splint applied to the right hand following simultaneous flexor and extensor tendon injury in Zones II and III. Color codes: Blue/green/yellow/orange (vertical): extensor tendon lines; orange (horizontal): metacarpophalangeal joint line; pink (horizontal): wrist joint line; black oval: hand contour; orange curve (lateral view): wrist flexion (0-15°).

Following surgery, the patient was referred to the Physical Medicine and Rehabilitation outpatient clinic for postoperative rehabilitation. Due to the complex nature of the injury and the combined involvement of flexor and extensor structures, a standard postoperative protocol could not be followed. Instead, the hand was immobilized in a mid-position using a custom static wrist-hand splint to protect all repaired tissues. The splint was applied with the wrist in neutral or slight flexion (0-15°), the metacarpophalangeal (MP) joints in 40-50° flexion, and the interphalangeal (IP) joints in slight flexion (0-10°) (Figure 1).

During the first postoperative week, circulation and wound healing were closely monitored, and no complications were observed. Immobilization was maintained for three weeks to protect the repaired structures. However, due to the high risk of adhesion formation in combined flexor and extensor tendon injuries, early controlled passive flexion and protected active extension exercises were initiated in accordance with previously reported rehabilitation principles emphasizing controlled mobilization to promote tendon gliding while minimizing rupture risk.

Progression from passive to active movement was based on predefined clinical criteria, including adequate wound healing, absence of excessive pain or edema, stable tendon repair on clinical examination, and the patient’s ability to perform passive movements without resistance or compensatory patterns.

From the third postoperative week onward, tendon gliding exercises and controlled active movements within a protected range were introduced. By the sixth week, the patient regained the ability to perform basic daily activities such as eating and writing, achieving limited yet functional hand use (Table 1).

**Table 1:** Rehabilitation protocol.

Period	Intervention	Objectives
0–3 weeks (protection phase)	Splinting, passive flexion, active extension exercises, edema control	Protect repairs, prevent adhesions
3–6 weeks (early mobilization phase)	Controlled active flexion–extension, tendon gliding exercises, light resistance exercises	Promote tendon gliding, prevent joint stiffness
6–12 weeks (functional phase)	Functional grip exercises, fine motor activities, desensitization training	Improve functional hand use, support sensory recovery
≥12 weeks (advanced phase)	Strengthening, coordination training, return-to-work program	Maximize functional capacity

Range of motion (ROM) measurements were performed using a standard finger goniometer by the same experienced clinician at each assessment to ensure consistency. Grip strength was evaluated using a hand-held dynamometer (Jamar-type), with measurements obtained in a standardized seated position with the elbow flexed at 90°, and the results were expressed as a percentage of the contralateral, unaffected hand to allow intra-patient comparison.

At baseline, ROM of the second and third fingers was limited, with the MP joints at 25%, proximal interphalangeal (PIP) joints at 20%, and distal interphalangeal (DIP) joints at 10% of normal motion. Grip strength was 15% of the contralateral hand. At week six, ROM improved to 65% in the MP joints, 55% in the PIP joints, and 45% in the DIP joints, with grip strength increasing to 45%. By week 12, further recovery was observed, with MP joints reaching 85%, PIP joints 80%, and DIP joints 75% of normal motion. Grip strength reached approximately 70% of the unaffected hand.

**Figure 2:**
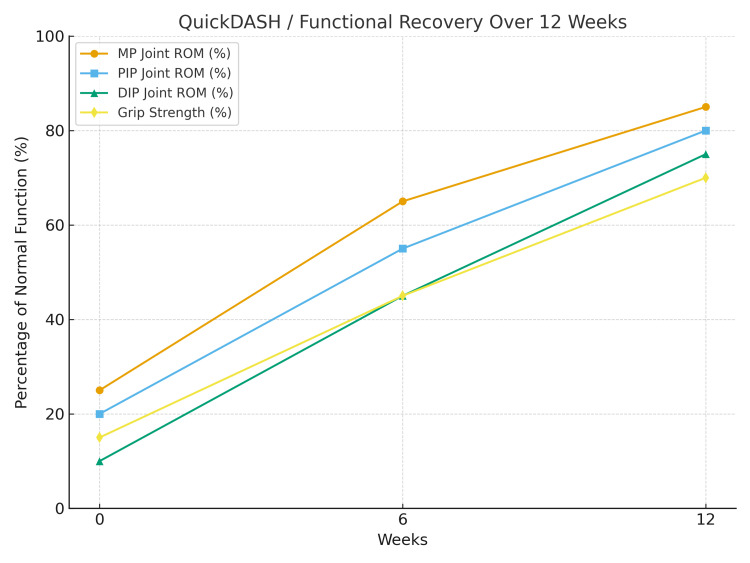
Functional recovery graph. QuickDASH: Quick Disabilities of the Arm, Shoulder, and Hand

Regarding median nerve involvement, manual muscle testing revealed progressive improvement in thenar muscle strength, increasing from grade 2/5 at baseline to 4/5 at week 12. The patient achieved independence in fine motor activities, including buttoning clothes, writing, utensil use, and handling small objects (Figure 2).

## Discussion

Complex hand injuries involving simultaneous damage to tendons, nerves, and vascular structures can lead to significant functional and aesthetic deficits. In such cases, postoperative rehabilitation is as crucial as surgical repair in determining long-term functional outcomes [2]. The literature on flexor tendon injuries has consistently demonstrated, through prospective studies and systematic reviews, that early controlled mobilization reduces adhesion risk and improves functional results. Cochrane reviews have also shown that early active motion protocols following flexor tendon repair provide superior ROM and functional outcomes compared to prolonged immobilization [7].

Extensor tendon repairs present additional challenges due to the superficial course of the tendons and the thin dorsal skin envelope, which increases vulnerability to adhesions and stiffness. Recent studies have demonstrated that early active mobilization protocols can also yield favorable functional outcomes following extensor tendon repair when applied in a controlled manner [8]. When flexor and extensor tendon injuries occur simultaneously, the rehabilitation strategy becomes even more complex, as multiple tendon planes and joint segments are involved.

In cases involving simultaneous flexor and extensor tendon repair, prolonged immobilization may increase the risk of joint stiffness and adhesion formation across multiple tendon planes. Therefore, carefully controlled early mobilization, tailored to protect all repaired structures, represents a rational strategy to balance tendon healing with functional recovery [9].

However, the presence of concomitant nerve repair necessitates additional caution, as excessive tension or premature loading may compromise neural healing. For this reason, rehabilitation protocols in such cases should be individualized, with gradual progression based on tissue healing, clinical examination, and functional tolerance rather than rigid timelines [4]. Similarly, when arterial repair is performed, the early rehabilitation phase should prioritize the preservation of vascular patency, edema control, and wound healing. During this period, cold applications should be avoided, and limb elevation with pressure-free bandaging is recommended to support circulation [10]. Successful vascular repair creates an optimal biological environment for subsequent tendon and nerve healing.

Overall, this case demonstrates that with careful planning, close monitoring, and a multidisciplinary approach, early and controlled rehabilitation can be safely implemented even after complex simultaneous repairs, contributing to meaningful functional recovery.

## Conclusions

In the presented case, a staged, controlled, and individualized rehabilitation protocol was implemented following simultaneous repair of complex structures. The process began with early passive exercises and progressed to protected active movements, sensory re-education, and strengthening exercises. This approach contributed to the preservation of tendon integrity and supported functional recovery during the early postoperative period. However, as this report reflects the outcome of a single patient with a relatively short follow-up period, conclusions regarding long-term nerve regeneration and full functional recovery should be interpreted with caution. While meaningful improvements in ROM, strength, and daily hand use were observed, sensory recovery and neural regeneration are expected to continue over a longer timeframe. This case highlights the potential role of early, balanced, and patient-specific rehabilitation in complex hand injuries, while underscoring the need for further studies with longer follow-up and validated outcome measures.
